# From diffusion in compartmentalized media to non-Gaussian random walks

**DOI:** 10.1038/s41598-021-83364-0

**Published:** 2021-03-03

**Authors:** Jakub Ślęzak, Stanislav Burov

**Affiliations:** Physics Department, Bar-Ilan University, Ramat Gan, 5290002 Israel

**Keywords:** Membrane biophysics, Single-molecule biophysics, Statistical physics

## Abstract

In this work we establish a link between two different phenomena that were studied in a large and growing number of biological, composite and soft media: the diffusion in compartmentalized environment and the non-Gaussian diffusion that exhibits linear or power-law growth of the mean square displacement joined by the exponential shape of the positional probability density. We explore a microscopic model that gives rise to transient confinement, similar to the one observed for *hop-diffusion* on top of a cellular membrane. The compartmentalization of the media is achieved by introducing randomly placed, identical barriers. Using this model of a heterogeneous medium we derive a general class of random walks with simple jump rules that are dictated by the geometry of the compartments. Exponential decay of positional probability density is observed and we also quantify the significant decrease of the long time diffusion constant. Our results suggest that the observed exponential decay is a general feature of the transient regime in compartmentalized media.

## Introduction

The Brownian motion is ubiquitous in applications, be it the microscopic motion of molecules^1^, search patterns of animals^2^, or the prices of financial options^3^. Following the classical argument of Einstein^4^, if there exists a time scale in which the changes of the investigated variable can be treated as a result of additive, independent and homogenous fluctuations with finite second moment, then in larger time scales the observed process is the Brownian motion. This insight was later formalised as the functional central limit theorem^5^.

The Brownian motion became a natural start for theoretical and experimental investigations of more complicated stochastic models. For example, lowering the requirement of finite moments led to the rich theory of Lévy flights^6^; lowering the assumption of independence was one of the cornerstones of the anomalous diffusion modelling^7^. The Langevin theory of diffusion investigates the time scales lower than those required by the central limit theorem^8^ which leads to the motions in which the deviations from Brownian motion appear in the memory structure; the probability density of motion is still Gaussian, only with a different scale.

In recent years the influx of experimental data proved the existence of a robust class of systems exhibiting non-Gaussian, in particular exponential, tails of the probability density^9–11^, together with normal or anomalous mean square displacement^12–14^. This common phenomenon is called *Brownian, yet non-Gaussian diffusion*^9,10^. Its raising prominence and importance in understanding the biochemical nature of the transport stimulated various attempts to provide some – at least effective – description. These include works using variants of the Langevin equation that use superstatististical^15–18^ or diffusing diffusivity^19–21^ approach.

**Figure 1:**
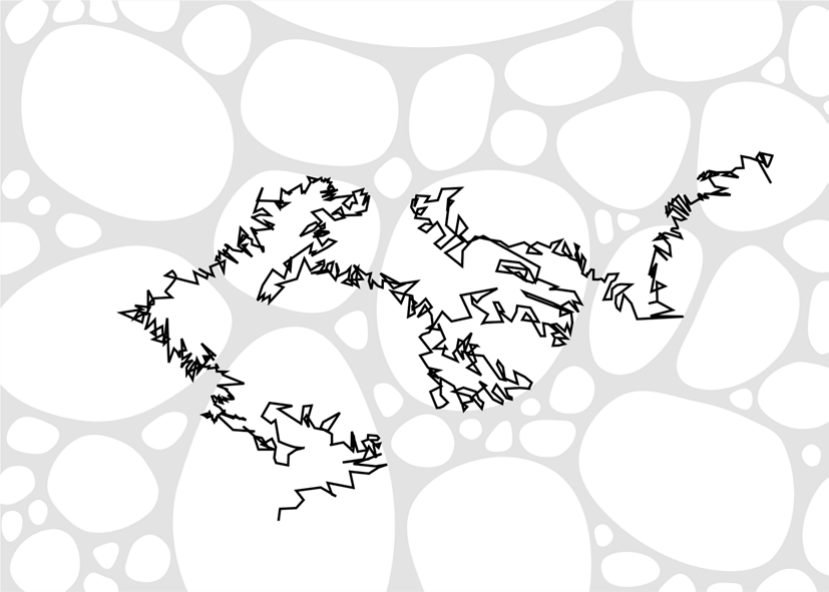
A schematic representation of the diffusion in a two dimensional compartmentalized medium. A particle (black trajectory) diffuses freely inside the domains (white areas) but only rarely crosses the thick divisions between them (grey area) which are thin compared to the average size of the domains and act as barriers.

It is commonly suspected that the true source of non-Gaussianity lays in the heterogeneity of the medium^9,21–23^. It is also hypothesised that the correct description may be related to random walks with traps^24,25^. Yet, formulating and solving suitable models is still a challenge far from completion. Here, we attempt to overcome it for systems in which the diffusion is locally impeded by barriers, providing a general mechanism for the appearance of the non-Gaussian diffusion; for a simplified illustration of the type of medium considered see Fig. 1.

Such restrictions of the molecular motions are ubiquitous in nature^26^, especially in composite or porous materials^27,28^. For example, according to the fences and pickets model the cellular membrane is compartmentalized (likely by actin-based membrane skeleton and various transmembrane proteins^29,30^), a fact essential in understanding its structure and functions^31,32^. It is also widely agreed that this phenomenon is crucial in determining the material properties of the biological media, the transport of proteins, lipids and their functions^31,33–35^.

The *transient confinement* caused by these obstacles should result in a motion similar to a random walk, with a particle jumping between adjacent domains. This phenomenon was experimentally observed and termed *hop diffusion*^36,37^. Its characteristic property is that the long-time macroscopic *effective diffusion coefficient* is greatly reduced compared to the short-time in-compartment diffusion coefficient with their ratio providing important insight into the molecular properties of the system. This ratio was found to be between 10 and 100 in various experiments, see the list in^38^. Similar results were found for porous materials^39–41^ and for colloidal particles near the glass transition; in the last case what they call the *cage effect* was observed together with the exponential tails of the probability density^42–44^.

In what follows, we first make a universal observation on how the confinement results in the static form of non-Gaussianity (“Non-gaussianity stemming from confinement” section). The argument is quite general and shows the time scales at which the increments of the diffusing particle’s position exhibit exponential tails. It does not specify the dynamics, but provides a single jump’s distribution, which is a basic building block of the random walk models which we derive. We study them using one dimensional model of the system. We start from reducing the local diffusivity model to the diffusion equation with interface conditions (“Transiently confined diffusion with locally Brownian dynamics” section), which we solve domain-wise in order to derive the transition times of the jumps between domains. Consequently, we manage to describe the diffusion using environment-dependent random walk (“From the transient confinement to a random walk” section). It can be effectively approximated using much simpler model of continuous time random walk (CTRW), yielding the formula which links the effective diffusion coefficient to the barriers’ permeability (“Bessel and gamma waiting times CTRWS” section). Next, we decouple the correlations of this process and reveal its broad-tail non-Gaussian nature using the kurtosis and the logarithm of characteristic function (“Compound poisson approximation” section). Finally, we consider a more reductive but mathematically elegant approximating CTRW for which we calculate the exact probability density; it exhibits exponential tails characteristic for the Brownian, yet non-Gaussian diffusion. The derivations presented in “Bessel and gamma waiting times CTRWS” and Compound poisson approximation” sections. show the mathematical origin of the analytical predictions presented in Figs. 8-11, but for general understanding of the results the form of the reduced model (14) and the leading exponential-tail behaviour (36) suffice. The overview of the subject and the results is given in “Discussion” section. We also provide a list of commonly used notation. Our simulation code is attached in the supplemental material.

## Non-Gaussianity stemming from confinement

A possible approach for understanding the prevalence of the Gaussian distribution is to look at it through the notion of entropy. For all different variables *X* in free space with a given variance, characterised by the probability density function (PDF) $p_X$, the ones with Gaussian distributions (with different means) maximise the entropy $-{\langle {\ln p_X(X)}\rangle }$. Thus, if they are no other constrains (such as potential field), any coordinate coupled to a heat reservoir will converge to a Gaussian state^45^.

This naturally opens up the question in what conditions the Gaussianity is *not* to be expected. Following the entropic argument, the simplest such case is any bounded domain in which the entropy is maximized by the uniform, not Gaussian distribution. Physically speaking, it corresponds to particles being confined in some finite area by a reflecting barrier. Such a perfect local confinement excludes the diffusion in the macroscopic scale, however in a more detailed setting one can think about a system with dynamics dominated by two time scales: the relaxation time $t_r$ of reaching the domain-wise stationary uniform state, and the average escape time $t_e\gg t_r$ of passing through the barrier. In this setting, for $t\ll t_r$ the scale of the dynamics is too small to be affected by the confinement. For $t\gg t_e$ as long as there are no “hard” traps it is expected that the system will be again homogenised and Brownian (even for the cases when the intra-domain dynamics was not, e.g. was subdiffusive). For the time range in-between, the motion is dominated by the confinement in one ($t\approx t_r$) or few ($t\approx t_e$) domains and always non-Gaussian. This time scale will be in the centre of our discussion.

For $t<t_e$ the situation is simple: for a given particle its distribution will be a uniform one with the shape reflecting the domain it was found in. For the whole ensemble the distribution will be a mixture of uniform distributions reflecting the domain shapes all over the medium. As we can see we are in the situation in which the observed ensemble distribution is non-Gaussian and completely determined by the random geometry of the system, to some extent independently form the details of the dynamics.

This geometry is clearly hard to determine for many real systems and may be very complex, e.g. porous media are often fractal-like. However, in many media such as cellar membranes it is reasonable to assume that the domains are mostly spherical and they vary mostly in size. Furthermore, the simplest size distribution to consider is the exponential one. The argument for this is the lack of dependence. We consider one dimensional line in the medium and ask if in any given ${\mathrm{d}}x$ it crosses the barrier or not. If the crossing in ${\mathrm{d}}x$ does not affect the distribution of other crossings (they do not “see” each other) they must be located according to the Poisson point measure^46^ and the distances between any two subsequent barriers on this line are exponential.

We are now ready to calculate the stationary PDF which will be observed for the whole ensemble. Let *L* denote the diameter of a given domain. By choosing proper displacement units we can always assume that ${\langle {L}\rangle }=1$ and its distribution of the sizes is a simple $p_L(l)=\exp (-l)$. However, this is not the proper PDF to average over as we need to account for the average number of particles in each domain. If the domains are not correlated to any kind of a trap, this number will be proportional to the volume of the domain. This leads to the equilibrium PDF^47^ $p_L^\text {eq}(l)=\ c_d l^d\exp (-l), c_d =1,1/2,1/6$ in dimensions $d=1,2,3$ respectively (these are gamma distributions $\mathcal {G}am(d+1,1)$, see the Notation section).

For a given domain with a fixed *L* the distribution is uniform, for one dimension it is a simple $p_X(x|L)=1/L, |x|<L/2$, which yields

1 $$\begin{aligned} p_X(x) = \int _{|x|<l/2} \!\!\!{\mathrm{d}}l \frac{1}{l}p^\text {eq}_L(l) = \int _{2|x|}^\infty \!\!{\mathrm{d}}l\mathrm {e}^{-l} = \mathrm {e}^{-2|x|}. \end{aligned}$$

**Figure 2:**
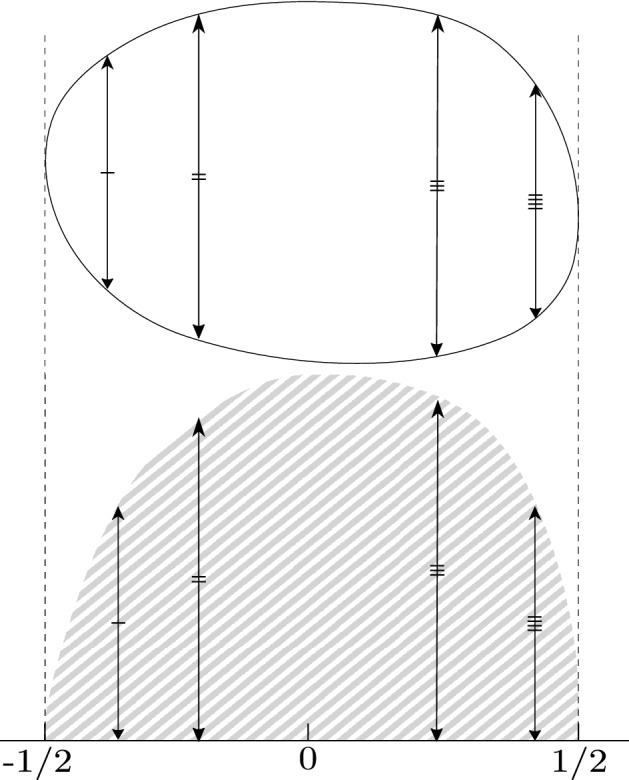
Projection procedure applied to an exemplary domain. All the probability mass in two dimensions (the oval shape up) is projected onto a given axis, i.e. moved down leaving lengths of the depicted arrows intact, resulting in one dimensional mass (schematically shown as grey area below); then it is renormalized.

This simple argument shows how the geometry of the system determines the distribution of *L* and is reflected in the stationary PDF of particles’ displacements, which is found to be the Laplace (also called “two sided exponential”) distribution with mean 0 and variance 1/2, symbolically ${\mathcal {L}}ap(0,1/2)$. For two and more dimensions the procedure is slightly more complicated: for each coordinate *x*, *y* or *z* we will observe only the projection of the total multidimensional probability mass, see Fig. 2. For spheres these are semicircular law for two dimensions, and parabolic law for three dimensions (as for each ${\mathrm{d}}x$ we squeeze onto it a circle with surface $\pi ((1/2)^2-x^2)$). The projections and averaged PDFs are shown in the first and second row of Table 1.

**Table 1:** Confined one-dimensional PDFs for the particles trapped in 1, 2, 3D spheres.

	1D	2D	3D
$p_X(x|L)$	$\frac{1}{L}$	$\frac{4}{\pi L}\sqrt{1-\left( \frac{2x}{L}\right) ^2}$	$\frac{3}{2L}\left( 1-\left( \frac{2x}{L}\right) ^2\right)$
$p_X(x)$	$\mathrm {e}^{-2|x|}$	$\frac{4|x|}{\pi }K_1(2|x|)$	$\frac{1}{4}(1+2|x|)\mathrm {e}^{-2|x|}$
$p_{\Delta X}(x)$	$\sim \mathrm {e}^{-|x|}$	$\sim \frac{8}{3\pi }\mathrm {e}^{-|x|}$	$\sim \frac{6}{|x|}\mathrm {e}^{-|x|}$

The conditional (i.e. with fixed trap diameter *L*) PDFs $p_X(x|L)$ in the top row are Wigner *n*-sphere distributions. Middle and bottom rows are the PDFs averaged over the trap sizes, of the displacement *X* and the increments $\Delta X := X_{t+\Delta t}-X_t, \Delta t\gg t_r$ respectively. By $K_1$ we denote the modified Bessel function of the second kind (see^48^, Eq. 10.32.8]) which has tails $K_1(2|x|)\sim ({\pi /x)^{1/2}} \exp (-2|x|)/2$. In the bottom row we show only the asymptotics, but in the case of 1D and 3D the exact values of $p_{\Delta X}$ can be calculated by direct integration, the formulas are complicated but may be expressed using special function Ei and polynomials.

The important thing to notice there is that they all decay like $\exp (-2|x|)$ multiplied by some power-law factor. Integrating by parts shows that this a more universal behaviour, the requirements for its occurrence are only that the PDF of the sizes has a tail $\propto l^\alpha \exp (-l)$ and the stationary PDF within a fixed domain has a cut-off at $\pm L/2$. However, there is a simplifying assumption in (1) which we did not immediately discuss: the centre of the domain is located at $x=0$, whereas in typical experimental data the position of the particle at the start of the measurement is taken as 0. This can be fixed with a litte effort and the corrected PDF still has exponential tails, see the Ref. ^49^.

The limitation of the practical usefulness of the insights made above is that the stationary distributions are visible in experiments only if many data points within the range $t_r<t<t_e$ can be measured. However, we can extend our approach if the sampling rate of the measurements is sufficiently smaller than the escape time, $t_r\ll \Delta t < t_e$. Because $\Delta t<t_e$ the subsequent values $X_t$ and $X_{t+\Delta t}$ will most often still be inside the same domain. Consequently, the series of increments $\Delta X_t :=X_{t+\Delta t}-X_t$ will consist of long intervals of the in-domain differences only rarely interrupted by the jumps to the adjoining domains; these rare spikes can be then neglected. Because $t_r\ll \Delta t$, the values $X_t$ and $X_{t+\Delta t}$ will be independent (as the particle will transverse the domain multiple times during $\Delta t$), so its PDF will determined by the difference of two independent variables with $p_X(x|L)$ distribution: this is triangular distribution in one dimension, complicated but elementary polynomial distribution in three dimensions and a rather involved one in two dimensions (this is because the convolution of two $\sqrt{1-x^2}$ functions is not elementary). In any case their tails can be derived using integration by parts, see the third row of Table 1. Using the ergodicity of $\Delta X_t$, this stationary distribution can be estimated using even a single long trajectory, no ensemble averaging is required. For all the cases this PDF also exhibits exponentially decaying tails.

The limit of this line of arguments is the time scale $t>t_e$ in which the dynamics becomes important. We need to know how long it takes for the particle to jump from one domain to another and for this a more detailed model of the barriers is required. This is the subject of the following sections.

## Transiently confined diffusion with locally Brownian dynamics

We imagine the heterogeneous medium as the collection of domains with regular shapes inside which the particle moves relatively freely, separated by narrow structures composed of a thick material (e.g. actin meshwork in the case of plasma membranes^29–32^) which impedes the diffusion within (these are grey areas in Fig. 1). We model this behaviour by making the diffusion coefficient locally small, which results in the stochastic differential equation

2 $$\begin{aligned} {\mathrm{d}}X_t = \sqrt{2D(X_t)}{\mathrm{d}}B_t. \end{aligned}$$

governed by the Brownian increments ${\mathrm{d}}B_t$. The local diffusivity function *D*(*x*) appearing here is a combination of two microscopic kinematic parameters: mean free path and correlation time. As such, the equation by itself is physically ambiguous and requires interpretation. It was previously established that if *D*(*x*) is to describe an environment with barriers (and no traps) present, the proper choice is *kinetic Hänggi–Klimontovich interpretation* in which the PDF of the diffusion solves the Fokker-Planck equation^50^

3 $$\begin{aligned} \frac{\partial }{{{\partial }t}}p_X(x;t) = \nabla \cdot \big ( D(x) \nabla p_X(x;t)\big ). \end{aligned}$$

In a bounded domain with volume *V* the constant PDF $p_X(x)=1/V$ is the unique stationary solution ($\partial p_X/\partial t=0$) of this equation, so this, and only this, interpretation agrees with our assumption of “no other constraints” in the medium and consequently leads to the maximal entropy distribution being uniform.

Up to this moment our considerations were quite general, from now on we will limit ourselves to the one dimensional system, which will allow us to simplify the geometry immensely and consequently obtain quite straightforward description of the dynamics. In one dimension the barriers are thin $\Delta x$ intervals with a small local diffusivity $D_b\ll D$. Let a barrier be a layer starting at $x_k$ and ending at $x_k+\Delta x$. Preservation of the number of particles forces the flux $D(x)\partial p_X/\partial x$ to be a continuous function, in particular at the borders of the barrier

4 $$\begin{aligned}&D\frac{\partial }{{{\partial }x}}p_X(x_k^-;t) = D_b\frac{\partial }{{{\partial }x}}p_X(x_k^+;t),\nonumber \\&D_b\frac{\partial }{{{\partial }x}}p_X(x_k+\Delta x^-;t) = D\frac{\partial }{{{\partial }x}}p_X(x_k+\Delta x^+;t). \end{aligned}$$

When $D_b\ll D$ these conditions cause $p_X$ to change rapidly inside the barrier, though this function must still be continuous. For the flux to be also continuous the derivative $\partial p_X/\partial x$ must be discontinuous at *x* and $x+\Delta x$, but with $p_X$ still being smooth inside the barrier. Now, we may express $p_X$ on the outside edges of barrier using the values inside, then make a Taylor approximation and apply (4)

5 $$\begin{aligned}&p_X(x_k+\Delta x^+;t) - p_X(x_k^-;t) \nonumber \\&\quad = p_X(x_k+\Delta x^-;t) - p_X(x_k^+;t) \nonumber \\&\quad = \Delta x \frac{\partial }{{{\partial }x}}p_X(x_k^+;t) + {\mathcal{O}} {\left( {\Delta x^2}\right) }\nonumber \\&\quad = D \frac{\Delta x}{D_b}\frac{\partial }{{{\partial }x}}p_X(x_k^+;t) +{\mathcal{O}} {\left( {\Delta x^2}\right) }. \end{aligned}$$

If we go to the limit $\Delta x\rightarrow 0$ and $D_b\rightarrow 0$ in such a way that $D_b/\Delta x \rightarrow \kappa D$ we end up with a diffusion determined by the heat equation within the domains

6 $$\begin{aligned} \frac{\partial }{{{\partial }t}}p_X(x;t) = D \frac{\partial ^{2}}{{{\partial }x}^2}p_X(x;t),\quad x\ne x_k \end{aligned}$$

which breaks at the locations of barriers where two *interface conditions* are linking the values of the PDF on their left and right side. The first one is again the flux continuity, the second one is the reduced form of (5)

7 $$\begin{aligned} \frac{\partial }{{{\partial }x}}p_X(x_k^-;t)&= \frac{\partial }{{{\partial }x}}p_X(x_k^+;t),\nonumber \\ \frac{\partial }{{{\partial }x}}p_X(x_k;t)&= \kappa \big (p_X(x_k^+;t)-p_X(x_k^-;t)\big ). \end{aligned}$$

The parameter $\kappa$ here is the barrier *permeability*; mind that some authors denote $\kappa ' = \kappa D$ as permeability instead. This argument can be generalized to two or three dimensional domains in a straightforward manner, see^51^. It is also worth adding that the same interface conditions can be derived using the barriers modelled by the bumps of potential, but the derivation is quite technical^52^. Mathematically speaking, this diffusion has a rather peculiar PDF: by making barriers’ thickness negligible we caused it to exhibit finite jump discontinuities at each $x_k$ but at the same time still has continuous directional derivatives everywhere. It is smooth only in the stationary state, which (in a finite space *V*) is uniform as required, $p_X(x;t\rightarrow \infty )=1/V$.

Conditions (7) also appear in chemistry^53,54^ and should come as no surprise: it is nothing but a stochastic version of the *Newton’s law of cooling*. In the most typical form it states that the heat flux at a boundary is proportional to the temperature difference, $\partial Q/\partial t \propto -(T_\text {in}-T_\text {out})$. Combining it with the Fourier’s law $\partial Q/\partial t \propto - \partial T_\text {in}/\partial x$ we end up exactly with (7). For this reason its commonly called “Newton” or “convection” boundary condition. However, our case is far less typical since we look for the solution on both sides of each barrier. This is in contrast to the thermal problems in which $T_\text {out}$ is most often taken to be a fixed state of the environment. For this reason the mathematical difficulty in solving the stochastic variant increases significantly.

**Figure 3:**
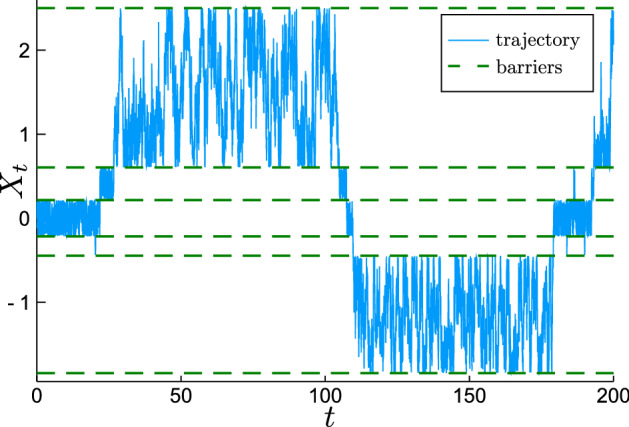
Exemplary simulated trajectory for $\kappa =1/50,D=1$ and discretization step $\Delta t = 0.01$. Barriers were located according to the standard Poisson random measure.

If only one barrier is present, let it be at $x_0=0$, the solution is still straightforward to obtain. One only needs to split the initial condition into symmetric and antisymmetric terms, $p_X(x;0) = p_X^S(x;0)+p_X^A(x;0)= (p_X(x;0)+p_X(-x;0))/2+ (p_X(x;0)-p_X(-x;0))/2$. Then the solution itself can be split into symmetric and antisymmetric terms which evolve independently with reflective (Neumann), $\partial p_X^S(0^+,t)/\partial x = 0$, and radiation (Robin), $\partial p_X^A(0^+,t)/\partial x = 2\kappa p_X^A(0^+,t)$ boundary conditions. Both can be solved using multiple standard methods. This argument also helps to understand physical meaning of the parameter $\kappa$. For $\kappa \rightarrow 0$ the boundary conditions converge to the purely reflective one and the particle stops being able to transverse the barrier. For $\kappa \rightarrow \infty$ they reduce to the condition $p_X^A(0^+;t)=0$. But as $p_X^A$ is antisymmetric by definition, it only forces $p_X^A$ to be a continuous function; the interface conditions and the barrier disappear altogether. The in-between case of a finite $\kappa$ is a partially reflective barrier or a *semi-permeable barrier*; look at Fig. 3 for an illustration how the resulting trajectories look like.

Alas, for any system with more than one barrier we loose this convenient symmetry^55^. The heat equation and interface conditions (7) are linear, but the dependence on the environment is non-linear, as expected for a heterogeneous system. For any finite number of barriers using the Fourier or Laplace transform in the position space reduces the problem to solving a system of algebraic equations, but the solutions are quite complicated and inverting the transform seems to be beyond reach for more than two barriers present.

Still, there exists a convenient stochastic representation of this diffusion which provides another layer of physical meaning and facilitates numerical simulations. In the mathematically impressive series of works Antoine Lejay derived such a representation for a system with one barrier^56–58^ which extends easily to the general case. If the space would be discrete the obvious and correct representation would be a random walk for which at each visit to the barrier there is a chance of passing or being reflected, see e.g.^59,60^. For continuous space this approach does not work directly because Brownian trajectories are so irregular they pass any threshold infinitely many times. The representation becomes valid if we restate this model as “each time the particle spends a unit of time at a barrier there is a chance of being reflected”. Now, as we decrease the discretization mesh the occupation times converge to the *local time* random field $\ell _t(x) :=\int _0^t{\mathrm{d}}\tau {\varvec{1}}_{X_\tau }(x)$^61^ (this is basically a histogram calculated from the sample trajectory $\{X_t\}$). At the same time the discrete escape times which have geometric distribution converge to the smooth exponential variables.

Indeed, Lejay showed that if we take a series of independent and identically distributed (i.i.d.) random times $\tau _1,\tau _2,\ldots \overset{d}{=}{\mathcal {E}}xp(\kappa )$ the process which is the reflected Brownian motion on the one side of the barrier until $\ell _t(x_0)>\tau _1$, then the reflected Brownian motion on the other side until $\ell _t(x_0)>\tau _1+\tau _2$ and so on, has a PDF which solves (6) and (7). This makes sense if we come back to the derivation: for a barrier with a finite thickness $\Delta x$ the particle must explore the inside of the barrier for the time long enough it will reach the other side. For small $\Delta x$ it is no surprise the passing is a Markovian event, thus the waiting time must be exponential.

The joint distribution of the reflected Brownian motion and its local time is known, so the condition $\ell _t(x)>\tau _1$ can be directly implemented in a stochastic simulation^57^. For multiple barriers system only the one on the left and the one on the right side of the current domain are important. Because the dynamics is Markovian and local, at each step it is only required to look for the closest barrier and check if the particle escaped through it in a given $\Delta t$ or not. This is the method used to simulate the trajectory shown in Fig. 3.

## From the transient confinement to a random walk

It is natural to suspect that the diffusion dominated by the transient confinement is a type of random walk, which is even suggested by the term “hop diffusion”. We will derive the random walk which corresponds to the model of the medium described in "Non-Gaussianity stemming from confinement". Similar approach was used as a possible explanation for Lévy flights^62^ in quenched disordered media^63,64^. However, in contrast to our system, for these phenomena the short scale motion is assumed to be ballistic and the distances between barriers have a power law distribution.

In our case, the process can be imagined as a particle which at each given time is localised according to a uniform distribution in the domain it is in, but after waiting a random escape time it transitions to a uniform distribution in one of the adjacent domains with the cycle starting again.

Let us consider one such domain with ends at $\pm L/2$. The escape time is determined by the solution of the diffusion equation if we remove returns to the domain, that is, after the escape event $\ell _t(\pm L/2)> \tau _1^\pm$ took place the particle becomes absorbed and cannot return. This is equivalent of putting absorbing (Dirichlet) boundary conditions just outside the domain walls. By instantly drawing out all the probability mass outside the domain this procedure reduces the interface conditions (7) to the radiation boundary conditions

8 $$\begin{aligned} \frac{\partial }{{{\partial }x}}p_X((\pm L/2)^\mp ;t) = \mp \kappa p_X((\pm L/2)^\mp ;t). \end{aligned}$$

The resulting process is a type of *partially reflected Brownian motion*^65^.

Using again the equivalence to thermal problems, the dynamics can be described by two dimensionless parameters: the Fourier number $Dt/L^2$ regulating the ratio of diffusive to transport motion and the Biot number $\kappa _B = \kappa L$ describing the ratio of heat resistance inside to that of the surface. Systems with small Biot number have uniform temperature, which is precisely our assumption of the dominance of local equilibria, which can now be specified to be the requirement that $\kappa _B\ll 1$. There is a nuance here as *L* and thus $\kappa _B$ are domain-dependent, but as the distribution of *L* has short tails, extreme values of $\kappa _B$ are improbable and we may just require that the average Biot number is small, ${\langle {\kappa _B}\rangle }\ll 1$.

By rescaling *t* and *x* the equation can be reduced to the dimensionless

9 $$\begin{aligned}&\frac{\partial }{{{\partial }t}} u(x;t) = \frac{\partial ^{2}}{{{\partial }x}^2}u(x;t),\nonumber \\&\frac{\partial }{{{\partial }x}}u((\pm 1/2)^\mp ;t) = \mp \kappa _B u((\pm 1/2)^\mp ;t) \end{aligned}$$

which we expand into a series of eigenfunctions

10 $$\begin{aligned} u = \sum _{n=0}^\infty a_n \mathrm {e}^{-\lambda _n^2 t}\cos (\lambda _n x)+ \sum _{n=0}^\infty b_n \mathrm {e}^{-\beta _n^2 t}\sin (\beta _n x). \end{aligned}$$

The eigenvalues solve $\tan (\lambda _n/2) = \kappa _B/\lambda _n$ and $\cot (\beta _n/2) = -\kappa _B/\beta _n$. For small $\kappa _B$ we can expand the trigonometric functions around their zeros and obtain

11 $$\begin{aligned} \lambda _n&= 2n\pi +\frac{2\kappa _B}{\sqrt{n^2\pi ^2+2\kappa _B}+n\pi }+{\mathcal{O}}{\left( {\kappa _B^2}\right) },\nonumber \\ \beta _n&= \lambda _n+ \pi ,\qquad n\in \{0,1,2,\ldots \}. \end{aligned}$$

**Figure 4:**
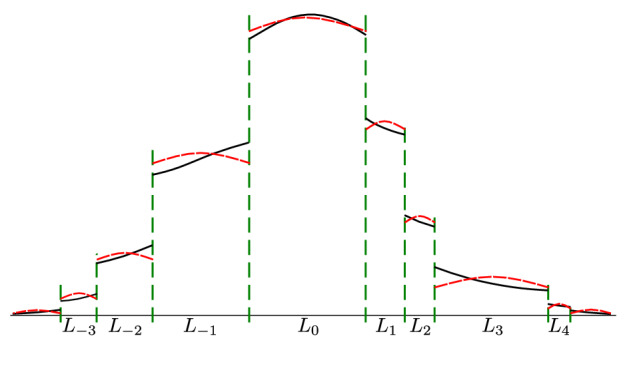
Schematic comparison of the original PDF (black lines) and the base mode approximation (red lines) with gradients exaggerated; for small $\kappa$ the functions are close to a step function with jumps at the barriers (green lines). The approximation has the shape $A_k(t)\cos (\sqrt{2\kappa /L_k}x)$ inside each domain, centred for the middle. The cosines are then approximated by ones, so the PDF is given by the amplitudes $A_k(t)$. The random walk approximation is equivalent to stating that $A_k$ evolve according to the master equation ${\mathrm{d}}A_k/{\mathrm{d}}t =2\kappa DL_k^{-1} (-A_k+A_{k-1}/2+A_{k+1}/2)$; one can also think about it as a trap model^66^, though with traps having varying sizes and exponential waiting times.

Due to our assumptions we expect the distribution to be mostly uniform, and it truly is: there is a spectral gap between the smallest eigenvalue $\lambda _0 = \sqrt{2\kappa _B}+{\mathcal{O}}{\left( {\kappa _B^2}\right) }$ and all the rest, thus the corresponding eigenfunctions decay much faster. After a brief relaxation only the mode $\cos (\lambda _0 x) = 1-{\left( {\kappa _B}\right) }$ remains and then it also decays with the slower rate $\exp (-2\kappa _B t)$. This is the PDF of the escape times for small $\kappa _B$. After coming back to the general case with all the physical constants explicitly present this distribution is found to be ${\mathcal {E}}xp(2 \kappa D /L)$. Observe that the average time spent in the domain during a visit is proportional to its length *L*, therefore this approximation preserves the globally uniform distribution of particles. We illustrate how the resulting approximation looks like for multiple domains in Fig. 4.

We are now able specify the random walk model. First, we need to fix the locations of the domains $x_k$. These are drawn independently for each trajectory which corresponds to a typical experiment where we trace multiple particles scattered across larger area.

**Figure 5:**
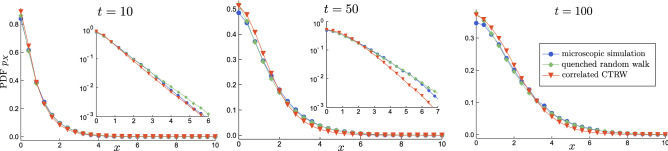
Comparison between the Euler scheme simulation ($\Delta t = 0.0005$) of the diffusion process (6), (7) with $D=1,\kappa = 1/50$, the random walk approximation $X_{2\kappa D t}$ in the quenched environment, and the annealed version with the correlated jumps and waiting times. (The PDFs are symmetrical, we only show the right half.) In the regime $t=10$ the mass contained in the initial domain is dominating; for $t=50$ the majority of the PDF corresponds to few jumps, $t=100$ is the start of the Gaussian regime (in the semi-log space they are nearly parabolas).

In "Non-Gaussianity stemming from confinement". we noted that if $x_k$ are independent from each other hey should be drawn from the stationary (i.e. translationally invariant) Poisson point measure. For simulations one can use the fact that the standard Poisson measure (corresponding to the first barrier put at point $x_0=0$) converges to the stationary one rather quickly. It suffices to put barriers one after another until sufficiently large *x* are reached (for domain size 1 few hundred is more than enough) and then move the beginning of the coordinate frame there. More elegant approach is to correct the initial domain. The proper choice was described in the Ref. ^49^, we draw $L_0\overset{d}{=}\mathcal {G}am(2,1), \Theta \overset{d}{=}{\mathcal {U}}nif(0,1)$ and put the left end at $(\Theta -1)L_0$, the right end at $\Theta L_0$. The rest of the domains is unaffected and they have lengths $L_k$ with i.i.d. ${\mathcal {E}}xp(1)$ distributions.

The random walk starts at domain $k=0$, after $T_1\overset{d}{=}{\mathcal {E}}xp(2 \kappa D /L_0)$ it jumps with probability 1/2 to $k=1$ domain or to $k=-1$ one. The jumping with the analogical domain-dependent waiting times repeats until we reach the final domain $k_f$ for which the sum of waiting times exceeds *t*. The position of the particle is then drawn from the uniform distribution ${\mathcal {U}}nif(x_{k_f},x_{k_f+1})$ where $x_k$ denotes the left end of the *k*th domain.

Conveniently, this approximation decouples parameters $\kappa$ and *D* from the dynamics, now they only determine the timescale. We may standardise this random walk by saying $X_t$ has ${\mathcal {E}}xp(1/L_k)$ waiting times, the physical units are can then be returned considering the process with rescaled time $X_{2\kappa D t}$. We will use this useful non-dimensional parameter further on, one can then return to our previous standard example with $\kappa = 1/50$ by dividing the time by the divisor 25.

This random walk depends on the local barriers’ placement (that is *quenched*) which makes the analytical analysis difficult. Both the domain sizes and waiting times are short-tailed distribution, so in this model the particle explores the space relatively freely and it may be expected that the annealing procedure should yield a good approximation. Actually, we will make another simplification at the same time by removing the correlation between the subsequent jumps and waiting times. For the quenched random walk each transition moves the probability mass into a uniform distribution inside some domain and is therefore correlated with the (domain size dependent) subsequent escape time. If we think about the quenched random walk as jumping from the middle to the middle of the domain, each transition has length $L_k/2+L_{k\pm 1}/2$. One can just consider annealed random walk with this property, but it can be considerately simplified if we just divide all the transition lengths in half.

The annealed model is thus as follows: we draw the initial domain size as $L_0\overset{d}{=}\mathcal {G}am(2,1)$ and the subsequent ones as i.i.d. $L_k\overset{d}{=}{\mathcal {E}}xp(1)$. The corresponding waiting times are drawn as $T_k\overset{d}{=}{\mathcal {E}}xp(2/L_k)$ or equivalently $T_k=E_k L_k/2, E_k\overset{d}{=}{\mathcal {E}}xp(1)$. Using them we construct a CTRW, i.e. the random sum $\sum _{k=0}^{N_t} J_k$ generated by jumps $J_k :=\pm L_k/2$ and the counting process $N_t :=\#\{k\colon T_1+\ldots + T_k\le t\}$. This process should approximate the centre of the domain the particle is in; to obtain the final position we account for the last local equilibrium by adding ${\mathcal {U}}nif((\Theta -1)L_0,\Theta L_0)$ if $N_t=0$ or ${\mathcal {U}}nif(-L_{N_t}/2,L_{N_t}/2)$ if $N_t\ge 1$.

We show the positional PDFs, averaged over independent numerical realisations of the environment and the particle, in Fig. 5. There we have chosen $D=1$ which determines the timescale and ${\langle {L_k}\rangle }=1$ which analogically fixes the position scale. The comparison proves that the random walk models are quite successful at capturing the bulk of the probability. There is a noticeable (but not huge) difference in the rate of exponential decay of the annealed process, but it is to be expected in this type of approximation.

The simulations also suggest that all the processes have similar Gaussian limit. For the annealed CTRW the Brownian limit can be derived in a formal way using the standard approach. We consider the rescaled process $X_{c t}/\sqrt{c}$ and then push the parameter *c* to infinity. In this limit the correction at the last site gets squeezed to zero, so it may be ignored. The rescaled counting process $N_{c t}/c$ converges to $t/{\langle {T_k}\rangle } = 2t$ (this is the renewal theorem) and the underlying classical random walk $S_n:=\sum _{k=0}^{n} J_k$ converges to the Brownian motion, $S_{cn}/\sqrt{c}\rightarrow \sqrt{{\langle {J_k^2}\rangle }}B_n = B_n/\sqrt{2}$. The CTRW $X_t$ is given by subordination of $S_n$ by $N_t$, so it converges to $B_{2t}/\sqrt{2}\overset{d}{=}B_t$^67^. Important thing to notice here is because the counting process $N_t$ collapses to a deterministic function, the dependence between waiting times $T_k$ and jumps $\pm L_k/2$ becomes irrelevant in the long time limit.

As a consequence, after accounting for all the physical constants, the long time effective diffusion coefficient $D_\text {eff} :=\lim _{t\rightarrow \infty } \delta _X^2(t)/(2t)$ of the annealed process is

12 $$\begin{aligned} D_\text {eff} = {\langle {\kappa _B}\rangle } D. \end{aligned}$$

**Figure 6:**
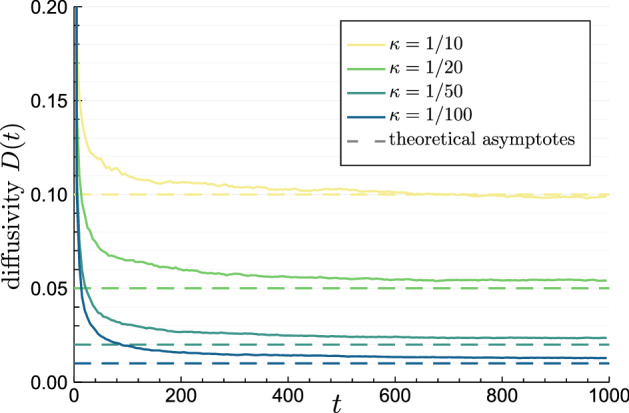
Time dependent diffusivity defined as $D(t) :=\delta _X^2(t)/(2t)$ calculated from the numerical simulation of the transiently confined process (6), (7) with short time $D=1$ and ${\langle {L_k}\rangle }=1$. For various permeabilities $\kappa$ the diffusivity *D*(*t*) starts at $D(0)=1$ and decays, reaching the level close to the value of theoretical prediction (12) derived for the annealed process. The range of $D_\text {eff}/D$ shown was suggested by the experiments of the hop diffusion^38^.

This agrees with the former results which were established for the systems with periodically placed barriers^39,68–71^. A comparison of $D_\text {eff}$ observed in the simulations of the transiently confined diffusion and the theoretical value (12) is shown in Fig. 6.

**Figure 7:**
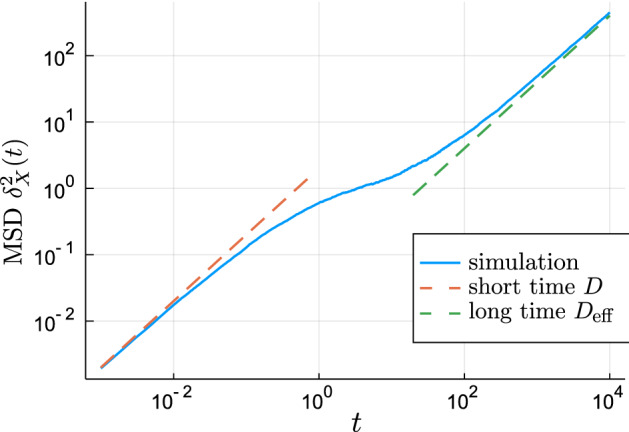
The MSD of the simulated transiently confined diffusion, $D=1,{\langle {L_k}\rangle }=1,\kappa = 1/50, \Delta t = 0.001$ (blue line) together with the short time prediction of the unconstrained Brownian motion with $\delta _X^2(t)\sim 2Dt$ (red dashed line) and the long time prediction (12) with $\delta _X^2(t)\sim 2\kappa Dt$ (green dashed line).

It is worth to note that this process is Fickian (has linear square displacement), but the transition from short-time diffusion constant *D* to the long-time $D_\text {eff}= {\langle {\kappa _B}\rangle }D$ causes the mean square displacement to be a convex function. This behaviour can be seen in Fig. 7 and bears great resemblance to contemporary experimental results for porous media^38,41^. The observed shape is easy to be mistaken for subdiffusion (MSD $\propto t^\alpha ,\alpha <1$); this “intermediate subdiffusive transport”^72^ suggest paying strong attention towards this possibility in the applied works as it could be misleading when observed without the entire six orders of magnitude shown on the time axis.

Figure 7 also indicates the time range in which we can expect the non-Gaussian behaviour. For short times the particle did not yet explore its initial domain and behaves like undisturbed Brownian motion with diffusivity *D*. For very long times the particle crossed a large number of domains, the environment becomes homogenised in this scale and the movements are again Brownian, but with lowered diffusivity $D_\text {eff}$. But, in the range between, where the MSD in Fig. 7 bends, the particle’s movements are strongly affected by the local environment, which originates the non-Gaussianity.

## Bessel and gamma waiting times CTRWs

It seems reasonable that the dependence between waiting times and jumps may not be a crucial aspect of the dynamics. We will remove it and analyse the resulting CTRW revealing its non-Gaussian behaviour.

For a particle exploring a medium with no long time correlation sources (such as strong traps) the dependence becomes irrelevant at long times and even at short times its is much weaker than in models such as Lévy walks. For the latter class, the amplitudes of jumps and waiting times are identical and the joint distribution is degenerate^73^. In our case it has a smooth PDF $p_{J,T}(x,\tau ) =\exp (-2|x|)\exp (-\tau /|x|)/|x|$ and the correlation between the jump amplitudes and waiting times has a lower value around 0.5.

Similarly, we may ignore the uniform distribution within the last domain and say that the particle just stops in the middle of the last domain. Using the same line of thought, we can say that the particle started in the middle of the first domain which therefore has form $[-L_0/2,L_0/2]$. Conveniently, it makes the distribution of the initial stationary state and of the subsequent jumps the same, that is the Laplace distribution ${\mathcal {L}}ap(0,1/2)$ derived in (1). It clearly introduces noticeable error for very short times when there is a significant probability mass in the initial domain. The uncorrelated CTRW PDF $p_X$ can be easily corrected by the formula

13 $$\begin{aligned} p_X'(x;t) = \mathrm{P}(T_1<t)p_X^\text {stat}(x;t) + \mathrm{P}(T_1\ge t)p_X(x;t), \end{aligned}$$

where $p_X^\text {stat}$ is the stationary PDF in the uncentred domain, see the Ref. ^49^. For the clarity of the presentation we will ignore this correction further on, but we note it makes the agreement shown in Fig. 10 better for the short times.

After all these steps, we end up with a CTRW

14 $$\begin{aligned} X_t = \sum _{k=0}^{N_t}J_k,\quad N_t=\#\{k:T_1+\cdots +T_k\le t\} \end{aligned}$$

with jumps $J_k\overset{d}{=}{\mathcal {L}}ap(0,1/2)$ and independent waiting times $T_k= E_k L_k/2$ . The sum starting from $k=0$ accounts for the initial distribution. Conditioning and direct integration shows that $T_k$ have PDF which can be expressed by the Bessel function, $p_T(\tau ) = 4 K_0(\sqrt{8\tau })$. This distribution or the distribution of $\sqrt{T_k}$ appear in various sources as the *Bessel distribution* or *K-distribution*^74,75^, we will use the former term. Its peculiar property is that this PDF has logarithmic singularity at $0^+$.

**Figure 8:**
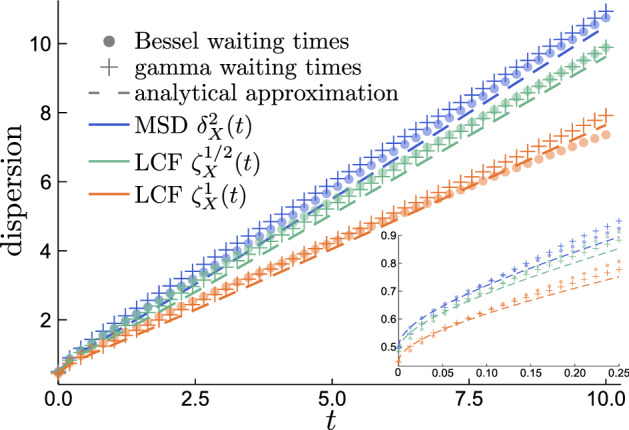
Comparison of the MSD and LCF with $\omega = 1/2,1$ for simulations of Bessel waiting times CTRW, gamma waiting times CTRW, and their analytical approximations (22) and (23). LCF being lower than MSD shows that the distribution has more spread out bulk than Gaussian.

The obtained Bessel waiting times CTRW is simple enough that one can use Montroll-Weiss formula to obtain expressions for the moments and PDF in Laplace and Fourier-Laplace spaces^7^. In particular it shows that the MSD has a logarithmic cusp $\propto t\ln (1/t)$ at small times $t\rightarrow 0^+$, see blue line in Fig. 8. However, we will not pursue this route, instead we will use a method which circumvents the use of Laplace transform and provides formulas which work globally with respect to *t*.

This is made possible if we approximate the waiting times distribution with a similar one for which the PDF of the counting process can be more easily managed. Counting processes with infinitely divisible waiting times are known to be well-studied; from this class short tailed processes are commonly modelled using gamma distribution (especially in finance^76^). From those, $\mathcal {G}am(1/2,1)$ is remarkably close to the Bessel PDF which is reflexed in very small Kolmogorov distance $\approx 0.0395$. To put this number in a perspective, in standard hypothesis testing setting, we would need samples with around 2000 observations to notice the difference. Crucially, this distribution also has the same mean, which is necessary to preserve the Brownian limit of the original process.

We remark that such a significant similarity seems to stem from deeper properties of the distributions in question. Gamma distribution has wrong both $x\rightarrow 0^+$ asymptotics ($1/\sqrt{x}$ instead of logarithmic) and $x\rightarrow \infty$ asymptotic (exponential instead of $\exp (-\sqrt{x})$). However, it nearly does not matter, as $\mathcal {G}am(\alpha ,1)$ for $\alpha = 1/2$ balances these two discrepancies so they cancel themselves out for the vast majority of the probability mass. The simple value of the parameter $\alpha = 1/2$ seems to be a coincidence, careful numerical study suggests that the value $\alpha \approx 0.494$ is marginally better. For any practical purpose the difference is insignificant but it is worth to add that the rest of the argument below does not depend on this particular value $\alpha =1/2$ but rather only on $\alpha$ being sufficiently close to 1.

We start with a counting process $N_t$ with ${\mathcal {G}}(1/2,1)$ waiting times. Knowing the exact distribution of the sums $T_1+\cdots +T_n$ we can determine the PDF of $N_t$ using the relation

15 $$\begin{aligned} \mathrm{P}(N_t \ge n)&= \mathrm{P}(T_1+\cdots + T_n \le t) \nonumber \\&= \mathrm{P}(\mathcal {G}am(n/2,1) \le t)= {\widetilde{\Gamma }}(n/2,t); \end{aligned}$$

here ${\widetilde{\Gamma }}$ is the regularised gamma function, ${\widetilde{\Gamma }}(a,t){:}=\int _t^\infty {\mathrm{d}}s s^{a-1}\exp (-s)/\Gamma (a)$. From this we get PDF expressed as a forward difference

16 $$\begin{aligned} p_N(n;t)&= \mathrm{P}(N_t = n) = \mathrm{P}(N_t \ge n) - \mathrm{P}(N_t \ge n+1)\nonumber \\&= {\widetilde{\Gamma }}(n/2,t) - {\widetilde{\Gamma }}((n+1)/2,t). \end{aligned}$$

The PDF of the whole CTRW can be linked to $p_N$ if we condition it by the number of jumps performed,

17 $$\begin{aligned} p_{X}(x;t) = \sum _{n=0}^\infty p_{S}(x;n) p_{N}(n), \end{aligned}$$

where $S_n$ is, as before, a partial sum process, $S_n :=J_0+\cdots +J_n$. To obtain its PDF we represent each jump as a difference of two independent exponential variables $J \overset{d}{=}{\mathcal {J}}^+-{\mathcal {J}}^-$, ${\mathcal {J}}^\pm \overset{d}{=}{\mathcal {E}}xp(2)$. Because of this their sum can also be split into $S_n = ({\mathcal {J}}_0^++\cdots +{\mathcal {J}}_n^+)-({\mathcal {J}}_0^-+\cdots +{\mathcal {J}}_n^-) = {\mathcal {S}}_n^+-{\mathcal {S}}_n^-$. Now, we can use the fact that ${\mathcal {S}}_n^\pm$ have a simple distribution, i.e. $\mathcal {G}am(n+1,2)$, and calculate $p_S$ as a convolution

18 $$\begin{aligned} p_{S}(x;n)&= \int _{-\infty }^\infty \!\!{\mathrm{d}}y p_{{\mathcal {S}}^+}(y;n)p_{{\mathcal {S}}^-}(-(|x|-y);n)\nonumber \\&= \int _0^\infty \!\!{\mathrm{d}}z p_{{\mathcal {S}}^+}(|x|+z;n) p_{{\mathcal {S}}^-}(z; n)\nonumber \\&= 4 \mathrm {e}^{-2|x|} \frac{4^n}{(n!)^2}\int _0^\infty \!\!{\mathrm{d}}z (z+|x|)^n z^n\mathrm {e}^{-4z}\nonumber \\&= \frac{1}{n!}\sum _{k=0}^n \frac{(2n-k)!}{k!(n-k)! 4^{n-k}} |x|^k \mathrm {e}^{-2|x|}. \end{aligned}$$

Finally, (16) and (18) substituted into (17) form the exact series representation of the PDF of CTRW with gamma waiting times and Laplace jumps. It can be easy plotted and compared with the data. Alas, because of the complicated form of (16) and (18) it is unwieldy for analytical investigation. To proceed, we will introduce another two approximations. The first yields the Fourier transform of the PDF and moments.

We will exploit the fact that the Poisson counting process has a simple PDF $t^n\exp (-t)/n!$ and survival function ${\widetilde{\Gamma }}(n,t)$. It has the same shape of graph as the distribution (15), i.e. ${\widetilde{\Gamma }}(n/2,t)$, as we see it is only rescaled by 1/2. So, for the PDF expressed as a forward difference between this function at $n+1$ and *n* we can replace the result by the Poisson PDF rescaled by 1/2

19 $$\begin{aligned} p_N(n;t)\approx \frac{1}{2}\frac{t^{n/2} }{\Gamma (n/2+1)} \mathrm {e}^{-t}. \end{aligned}$$

As mentioned previously, the same argument works also for more general ${\mathcal {G}}(\alpha ,\beta )$ waiting times for which the PDF would be rescaled by $\alpha$ instead of 1/2. What we did here is basically a perturbation of the counting process around the Poisson one which states that as long as the power law $t^{\alpha -1}$ at $t\rightarrow 0^+$ is not to far away from $\alpha =1$ the obtained distribution is the Poisson one rescaled.

This procedure does not preserve the normalisation of the PDF. Correcting it leads to

20 $$\begin{aligned} p_N(n;t)\approx \frac{t^{n/2} }{\Gamma (n/2+1)} \frac{1}{E_{1/2}\big (\sqrt{t}\big )}, \end{aligned}$$

where $E_{1/2}$ is the Mittag-Leffler function; for this particular parametrisation it can be also expressed as $E_{1/2}(x) = \exp (x^2){{\,\mathrm{erfc}\,}}(-x)$.

The error we make in this approximation is a differentiation error; it depends only on the smoothness of the underlying function. Therefore, it will become smaller when *t* increases and the distribution spreads. So for large times this correction of normalisation is unimportant, but it helps for the small times, making the approximation globally efficient.

To simplify the formulas, for the remainder of this section let us mark out the initial condition, i.e. decompose $X_t= {\mathcal {X}}_t + X_0$. By conditioning, we relate the distribution of ${\mathcal {X}}_t$ in the Fourier space to the transform of the jumps’ PDF ${\widehat{p}}_J(\omega ) = 1/(1+\omega ^2/4)$,

21 $$\begin{aligned}{\widehat{p}}_{{\mathcal {X}}}(\omega ;t) &= \sum _{n=0}^\infty \big ({\widehat{p}}_J(\omega )\big )^n p_{N}(n;t) \nonumber \\&\approx \sum _{n=0}^\infty \frac{z^{n}}{\Gamma (n/2+1)} \frac{1}{E_{1/2}\big (\sqrt{t}\big )}= \frac{ E_{1/2}(z)}{ E_{1/2}\big (\sqrt{t}\big )}, \end{aligned}$$

where for brevity we denoted $z:=\sqrt{t}/(1+\omega ^2/4)$. This formula can be directly compared against experimental data by using the sample estimate of ${\langle {\cos (\omega X_t)}\rangle }$.

We want to reveal the non-Gaussian nature of the process and a convenient form to do is log-characteristic function (LCF) $\zeta ^\omega _{\mathcal {X}}(t) :=-2\ln {\langle {\cos (\omega {\mathcal {X}}_t)}\rangle }/\omega ^2$. It measures dispersion of the displacements like MSD but gives bigger emphasis of the spread of probability bulk, so it is expected to be smaller than MSD for processes with a broader tails and more peaky PDF (for any $\omega$)^77^. Indeed, for

22 $$\begin{aligned} \zeta ^\omega _{\mathcal {X}}(t) \approx t\frac{1+\omega ^2/8}{(1+\omega ^2/4)^2} +\ln \frac{{{\,\mathrm{erfc}\,}}(-z)}{{{\,\mathrm{erfc}\,}}\big ({-\sqrt{t}}\big )} \end{aligned}$$

the first linear term is dominating for the large times and the second one $\sim \sqrt{t} \pi ^{-1/2}/(1+\omega ^2/4)$ is dominating for the short times; both have scaling coefficients smaller than those of the MSD, which we calculate as the second derivative of (21)

23 $$\begin{aligned} \delta ^2_{{\mathcal {X}}}(t)&= {\langle {{\mathcal {X}}_t^2}\rangle } = -\frac{\partial ^{2}}{{{\partial }\omega }^2} {\widehat{p}}_{{\mathcal {X}}}(\omega ; t)\Big |_{\omega = 0} \nonumber \\&\approx t +\pi ^{-1/2}\frac{\sqrt{t}}{{{\,\mathrm{erfc}\,}}\big ({-\sqrt{t}}\big )}\mathrm {e}^{-t}. \end{aligned}$$

Variable ${\mathcal {X}}_t$ corresponds to $X_0=0$ initial condition, but to account for the non-zero one we only need to add ${\langle {X_0^2}\rangle }=1/2$ to the MSD and $-2\ln {\langle {\cos (\omega X_0)}\rangle }/\omega ^2=2\ln (1+\omega ^2/4)/\omega ^2$ to the LCF (it is as expected always $\le 1/2$). For an illustration of the behaviour of these dispersion measures see Fig. 8. It is also interesting to note that the MSDs shown there prove that in practice it is very hard to see the difference between the logarithmic cusp (at $t\rightarrow 0^+$) of the Bessel waiting times CTRW and the square-root cusp of the gamma waiting times CTRW whereas the difference in asympotics might have suggested otherwise.

Continuing the analysis of non-Gaussianity, it can be also described using higher moments and one of particular interest is the excess kurtosis

24 $$\begin{aligned} {\mathcal {K}}_X(t) :=\frac{{\langle {X_t^4}\rangle }}{{\langle {X_t^2}\rangle }^2} - 3 = \frac{{\langle {{\mathcal {X}}_t^4}\rangle }-3{\langle {{\mathcal {X}}_t^2}\rangle }^2 + {\langle {X_0^4}\rangle }-3{\langle { X_0^2}\rangle }^2}{\big (\!{\langle {{\mathcal {X}}_t^2}\rangle }+{\langle {X_0^2}\rangle }\!\big )^2}. \end{aligned}$$

For any Gaussian variable it is 0; variables with tails broader than Gaussian are expected to have positive excess kurtosis (to be “leptokurtic”) and in particular for any Laplace variable it equals 3. We get the fourth moment from the fourth derivative,

25 $$\begin{aligned} {\langle {{\mathcal {X}}_t^4}\rangle }&= \frac{\partial ^{4}}{{{\partial }\omega }^4} {\widehat{p}}_{{\mathcal {X}}}(\omega ; t)\Big |_{\omega = 0} \nonumber \\&\approx t (t+9/2) + 3 \pi ^{-1/2}\frac{ (t+1) \sqrt{t}}{{{\,\mathrm{erfc}\,}}\big ({-\sqrt{t}}\big )}e^{-t}. \end{aligned}$$

**Figure 9:**
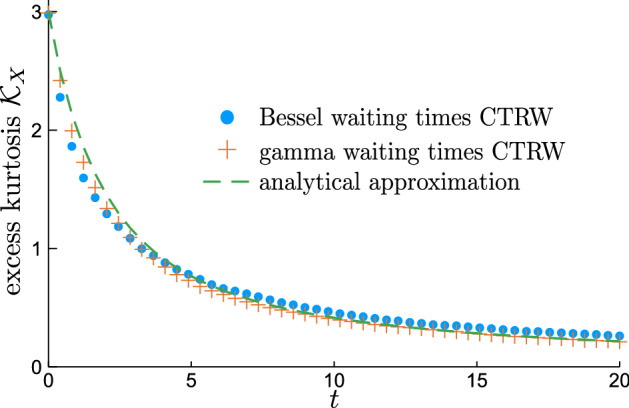
Excess kurtosis calculated from the simulations of CTRW with Bessel waiting times, gamma waiting times compared with the approximation (23),(25). This type of shape is shows relaxation from Laplace to Gaussian distribution.

Substituting the calculated averages into (24) leads to a rather bulky, but purely elementary formula for ${\mathcal {K}}_X(t)$, which is compared against simulations in Fig. 9. This function starts from ${\mathcal {K}}_X(0) = 3$ and then monotonically decays, at the beginning with rate ${\mathrm{d}}\mathcal {K}_X/{\mathrm{d}}t = 6(1-4/\pi )$ and as time grows the decrease becomes faster, reaching asymptotic $\sim 9/(2t)$. This behaviour reflects the initial Laplace regime which transitions to the long-time Gaussian relaxation.

As a side remark, we note that the kurtosis of ${\mathcal {X}}_t$ diverges at 0, ${\mathcal {K}}_{{\mathcal {X}}}(0^+) = \infty$ and then it converges to 0 in the same manner as ${\mathcal {K}}_X(t)$. This happens because kurtosis measures the broadness of the distribution’s tails and also its spikiness at $x=0$. In this case the latter is the culprit: the initial condition ${\mathcal {X}}_0 = 0$ causes the distribution of ${\mathcal {X}}_t$ to have Dirac delta at $x=0$ as ${\mathcal {X}}_t=0$ unless $T_1>t$. For a system like ours it is clearly an unphysical artefact, which shows the importance of a proper choice of the initial condition.

## Compound Poisson approximation

**Figure 10:**
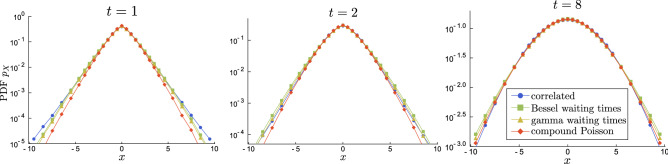
Comparison of the correlated CTRW and further approximations. Bessel and gamma waiting times CTRWs are nearly undistinguishable, all others are also close. These random walks have the same Gaussian limit, as shown for $t=8$. Due to using non-dimensional time in CTRW approximations, the plots here correspond to the same time regimes as in Fig. 5.

The methods which we used in the former section were successful in revealing the broad tails of the studied diffusion, but it would be beneficial to show the exponential decay of the PDF directly^78^. To achieve this we are introducing a second CTRW approximation with a very simple memory structure which will yield a full asymptotic series expansion of the PDF.

The insight that we use is a particular interpretation of the gamma waiting times CTRW. The PDF of the gamma waiting times process agrees with the Poisson counting process at even *n*. This is because a sum of any pair of $\mathcal {G}am(1/2,1)$ waiting times has exponential waiting time, $T_k+T_{k+1}\overset{d}{=}\mathcal {G}am(1/2,1)+\mathcal {G}am(1/2,1)\overset{d}{=}{\mathcal {E}}xp(1)$. Essentially, if we ignore odd numbers of jumps, the process behaves exactly like the one with exponential waiting times which makes double jump each time. Previously, we accounted for the odd numbers of jumps by interpolating the PDF with the Poisson formula.

The second possibility is to replace the counting process with double jumps by the one with twice the intensity but only single jumps. This way the average number of jumps remains the same (we just spread them), however we distort their variability. For this reason the second approximation is expected to be less accurate than the first one, but it makes up for it by providing useful representation of the PDF. For a comparison of this process to the other CTRWs considered in this work see Fig. 10, which proves that the approximation error mainly affects the low probability tails, whereas the bulk is preserved.

Thus, we have come to consider the CTRW with Laplace jumps and the Poisson counting process ${\mathcal {P}oiss}(2t)$. In the decomposition $X_t = {\mathcal {X}}_t+X_0$ the term ${\mathcal {X}}_t$ now belongs to the class of *compound Poisson processes*. These processes are very regular, being Markovian, infinitely divisible and having independent increments. As one of the consequences, ${\mathcal {X}}_t$ must have linear MSD, precisely $\delta ^2_{{\mathcal {X}}}(t) = t$. It means that in this approximation we completely neglect the non-linearity of the MDS present in the more detailed models. However even in those, the linear range was appearing quickly (even when the motion was still highly non-Gaussian), see Fig. 8, so the error caused by this is not huge.

The Fourier space representation of the compound Poisson PDF often has a sleek form; in our case

26 $$\begin{aligned} {\widehat{p}}_{{\mathcal {X}}}(\omega ; t) = \exp \left(2t\left( \frac{1}{1+\omega ^2/4}-1 \right) \right). \end{aligned}$$

Again, we may use it to calculate excess kurtosis, which is a simple rational function

27 $$\begin{aligned} {\mathcal {K}}_X(t) = \frac{3 t + 3/4}{(t+1/2)^2}. \end{aligned}$$

This function decays to 0 even slower than in the case of the gamma waiting times, ${\mathrm{d}}\mathcal {K}_X/{\mathrm{d}}t = 0$ at $t=0^+$ and the asymptotic decay is $\sim 3/t$, but overall the shape of this function is not much different than before.

Now, to get the PDF of $X_t$ in the position space we will use a particular representation available only for the compound Poisson processes. Instead of dividing each jump into the difference of two variables like before, we separate them into two categories corresponding to which are positive and which are negative for a given trajectory; the same is made to the initial condition. This can always be done, but only for the Poisson process the two thinned counting processes obtained, $N_t^+ :=\#\{k\colon T_1+\cdots +T_k, J_k>0\}$ and $N_t^- :=\#\{k\colon T_1+\cdots +T_k, J_k<0\}$ are independent and Poisson with the twice smaller intensity, $N_t^\pm \overset{d}{=}{\mathcal {P}oiss}(t)$ (this is a particular consequence of the process being Markovian). Thus, we may represent the total displacements as a difference of two positive, independent processes, $X_t = X_t^+-X_t^-$, where

28 $$\begin{aligned} X_t^\pm = \sum _{k=0}^{N_t^\pm } {\mathcal {J}}_k^\pm , \quad \text {i.i.d. } {\mathcal {J}}_k^\pm \overset{d}{=}|J_k| \overset{d}{=}{\mathcal {E}}xp(2). \end{aligned}$$

We again use the fact that ${\mathcal {J}}_0+\cdots + {\mathcal {J}}_n\overset{d}{=}\mathcal {G}am(n+1,2)$; conditioning by the number of jumps leads to a surprisingly elegant formula for the PDF of $X_t^\pm$^79^,

29 $$\begin{aligned} p_{X^\pm }(x;t)&= \sum _{n=0}^\infty p_{\mathcal {G}am(n+1,2)}(x) p_{N^\pm }(n;t)\nonumber \\&= 2\left( \sum _{n=0}^\infty \frac{(2xt)^n}{(n!)^2}\right) \mathrm {e}^{-2x}\mathrm {e}^{-t} = 2 I_0\big (2\sqrt{2t x}\big )\mathrm {e}^{-2x}\mathrm {e}^{-t}. \end{aligned}$$

From this we immediately determine the part of $p_X$ corresponding to the series of jumps in a one direction, these far tails are given by $\mathrm{P}(X_0>0)p_{N^-}(0;t)p_{X^+}(x;t) = I_0(2\sqrt{2t x})\mathrm {e}^{-2x}\mathrm {e}^{-2t}$. Of course the full PDF is significantly broader. As in (18) it is given by the convolution

30 $$\begin{aligned} p_X(x;t)&= \int _0^\infty \!\!{\mathrm{d}}z p_{X^+}(|x|+z;t) p_{X^-}(z; t)\nonumber \\&= \mathrm {e}^{-2|x|}\mathrm {e}^{-2t} \int _0^\infty \!\!{\mathrm{d}}z I_0\big (2\sqrt{2t (|x|+z)}\big )I_0\big (2\sqrt{2t z}\big )4\mathrm {e}^{-4z}. \end{aligned}$$

Let us denote the integral on the right in the above by ${\mathcal {I}} = {\mathcal {I}}(x;t)$. As Bessel function $I_0$ increases monotonically, but slower than exponentially, ${\mathcal {I}}(x;t)$ is also increases slower than exponentially with respect to *t* and *x*. The dominating factor of the tails is thus $\exp (-2|x|)$ which is completely static and is inherited from a single jump PDF, the rest is some time dependent factor and second order correction with respect to *x*. These are important as they contain information about the dynamics. The rest of the section is devoted to their derivation.

The integral ${\mathcal {I}}$ can be expanded into a series. One method is using Taylor expansion of $I_0$; this leads to a formula similar to the one shown in the previous section. However, more efficient expansion can be obtained if we integrate by parts.

It is easier to explain the general method first: under mild regularity assumptions on *f*, an integration with exponent can be expressed as the infinite order differentiation operator

31 $$\begin{aligned} \int _0^\infty \!\!{\mathrm{d}}z f(z) a\mathrm {e}^{-a z}&= f(0)+\int _0^\infty \!\!{\mathrm{d}}z \frac{\hbox {d}}{{\hbox {d}z}}f(z) \mathrm {e}^{-a z}\nonumber \\&= f(0)+ \frac{1}{a}\frac{\hbox {d}}{{\hbox {d}z}}f(z)\Big |_{z=0} + \frac{1}{a}\int _0^\infty \!\!{\mathrm{d}}z \frac{\hbox {d}^{2}}{{\hbox {d}z}^2}f(z) \mathrm {e}^{-a z} = \ldots \nonumber \\&= \sum _{n=0}^\infty \frac{1}{a^n}\frac{\hbox {d}^{n}}{{\hbox {d}z}^n}f(z)\Big |_{z=0} = \frac{1}{1-\frac{1}{a}\frac{\hbox {d}}{{\hbox {d}z}}} f(z) \Big |_{z=0}. \end{aligned}$$

In our specific case we need to take $f(z) = I_0\big (2\sqrt{2t (|x|+z)}\big )\times I_0\big (2\sqrt{2t z}\big )$ and $a= 4$. Function $I_0\big (\sqrt{z}\big )$ is absolutely monotonic, so the obtained series consists of positive terms, i.e. we divide the probability mass into Bessel-like modes.

It can be further simplified if one notes that the derivatives of $I_0\big (2\sqrt{2t z}\big )$ reduce to factors depending only on *t*. We expand each derivative using the binomial formula

32 $$\begin{aligned} \frac{\hbox {d}^{m}}{{\hbox {d}z}^m}\big (f(z)g(z)\big ) = \sum _{k=1}^m\left( {\begin{array}{c}m\\ k\end{array}}\right) \frac{\hbox {d}^{k}}{{\hbox {d}zk}^k}f(z) \frac{\hbox {d}^{{m-k}}}{{\hbox {d}z}^{m-k}}g(z) \end{aligned}$$

and then rearrange the terms according to the order of derivative acting on $I_0\big (2\sqrt{2t (|x|+z)}\big )$. The coefficient before the *n*th one is

33 $$\begin{aligned} & {} \sum _{k=n}^\infty \left( {\begin{array}{c}k\\ n\end{array}}\right) \frac{1}{4^{k}} \frac{\hbox {d}^{{k-n}}}{{\hbox {d}z}^{k-n}} I_0\big (2\sqrt{2t z}\big ) \Big |_{z=0}\!\!=\sum _{k=n}^\infty \left( {\begin{array}{c}k\\ n\end{array}}\right) \frac{1}{4^{k}} \frac{(2t)^{k-n}}{(k-n)!}\nonumber \\&\quad = \frac{1}{4^{n}} {}_1F_1(1+n,1;t/2) = \frac{1}{4^{n}}{\mathrm{e}}^{t/2}\sum _{k=0}^n \frac{(n-k+1)_k}{(k!)^2}\left( \frac{t}{2}\right) ^k, \end{aligned}$$

where $_1F_1$ is Kummer’s function and $(x)_k$ is Pochhammer symbol. Denoting the polynomial on the right by $q_n(t)$ the integral of interest takes the form

34 $$\begin{aligned} {\mathcal {I}}&= \mathrm {e}^{t/2} \sum _{n=0}^\infty \frac{q_n(t)}{4^{n}} \frac{\hbox {d}^{n}}{{\hbox {d}x}^n} I_0\big (2\sqrt{2t|x|}\big )\nonumber \\&= \mathrm {e}^{t/2} \sum _{n=0}^\infty \frac{q_n(t)}{4^{n}} \left( \frac{2 t}{|x|}\right) ^{n/2} I_n\big (2\sqrt{2t|x|}\big ). \end{aligned}$$

Alternatively, instead of using $I_n$, one can also repeatedly apply equalities ${\mathrm{d}}I_0(z)/{\mathrm{d}}z = I_1(z)$, ${\mathrm{d}}I_1(z)/{\mathrm{d}}x = I_0(z)- I_1(z)/z$ and express the result as a mixture of $I_0$ and $I_1$ multiplied by powers of *t* and *x*. In any case, the Bessel modes decay with respect to *n* and have prefactors of type $t^\alpha /x^\beta$, which shows that this expansion may converge slowly for large *t* and small *x* but will converge quickly for small *t* and large *x*.

Going back to the PDF under consideration, the expansion becomes

35 $$\begin{aligned} p_X(x;t)&= \sum _{n=0}^\infty \frac{q_n(t)}{4^{n}} \left( \frac{2 t}{|x|}\right) ^{n/2} I_n\big (2\sqrt{2t|x|}\big ) \mathrm {e}^{-2|x|}\mathrm {e}^{-3t/2}\nonumber \\&= I_0(2\sqrt{2t|x|}) \mathrm {e}^{-2|x|}\mathrm {e}^{-3t/2}\nonumber \\&\quad + \frac{1}{4}(1+t/2)\sqrt{\frac{2 t}{|x|}}I_1\big (2\sqrt{2t|x|}\big )\mathrm {e}^{-2|x|}\mathrm {e}^{-3 t/2} +\cdots \end{aligned}$$

**Figure 11:**
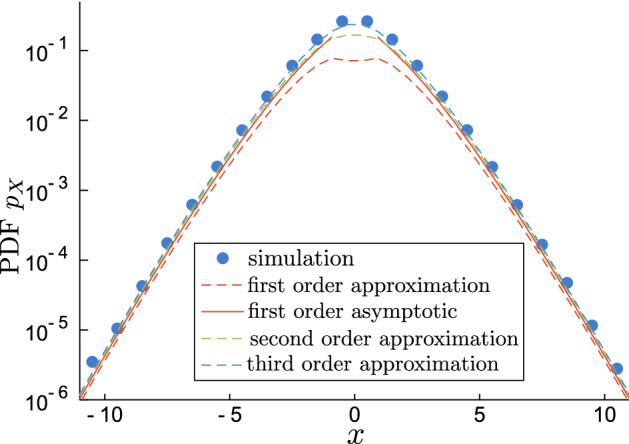
Comparison of the simulated compound Poisson process, expansion (35) and asymptotical formula (36) for $t=2$. For smaller times $t\lesssim 1$ the fit is even better. For larger *t*, as Gaussian regime starts the exponential behaviour is pushed towards larger |*x*|.

Using the tail asymptotic $I_n(z)\sim \mathrm {e}^{z}/\sqrt{2\pi z}$ we get the leading behaviour for large |*x*|

36 $$\begin{aligned} p_X(x;t)\sim \big (4\pi \sqrt{2t|x|}\big )^{-1/2}\mathrm {e}^{2\sqrt{2t|x|}}\mathrm {e}^{-2|x|}\mathrm {e}^{-3t/2} \end{aligned}$$

confirming the exponential decay of the PDF visible from the simulations, see Fig. 11 for a comparison.

Equation (36) is similar to the large deviation property. We have shown that $\ln p_X(x;t) \sim -t f(|x|/t)$ with the rate function $f(y) = 3/2+2y-2\sqrt{2y}$. This works for large |*x*| and any *t*, but for the larger *t* the convergence becomes progressively slower; this is caused by the coefficients $q_n(t)$ increasing with *t*. Nonetheless, it is not always necessarily the best formulation of the problem. For small *t* the rate function *f*(|*x*|/*t*) diverges, but we can still use Eq. (35) because it is exact. For example, if $|x|\rightarrow \infty$ and $t|x|\rightarrow \infty$ the result is the same, but if we go with $t\rightarrow 0$ fast enough such that $t|x|\rightarrow 0$ we get $\ln p_X(x;t)\sim 2t|x|-2|x|-3t/2$.

In any case, the most important factor is the exponential decay $\exp (-2|x|)\exp (-3t/2)$ which dominates the shape of the PDF in the semi-log scale. The rest are corrections which mostly move the straight line $-2|x|-3t/2$ around the coordinate frame. This non-Gaussian Laplace behaviour is observed in the “interim” regime in which the particle explores the neighbourhood of several domains and the effective diffusivity has already been reduced close to its long-time limit; it is the middle part of the range shown in Figs. 6 and 7 and middle plots in Figs. 5 and 10 . As a reminder, the results shown here are presented in the non-dimensional time *t* which is related to the physical time ${\tilde{t}}$ by scaling $t = 2\kappa D {\tilde{t}}$ as explained in "From the transient confinement to a random walk". The displacement unit is the mean distance between the barriers.

## Discussion

A robust and growing class of observations is exhibiting linear or power-law mean-square displacement joined by the pronouncedly non-Gaussian probability density, which most commonly has the exponential (Laplace) shape^9–14^ (see also the list of references in^21^). Explaining this phenomena related to *Brownian, yet non-Gaussian diffusion* is crucial for understanding transport within these media and their biochemical properties, a timely issue especially in biology and medicine. In spite of this demand, most of the studied analytical models are very case-specific^80,81^ or do not provide description which would reflect the real structure of the considered material, the most prominent examples being the superstatistical^15–18^ and the diffusing diffusivity^19–21,82^ approaches. The line of research presented here follows the widespread conviction that the physical origin of these observations lays in the heterogeneity of the studied media and try to emulate it by randomizing the parameters which appear in the dynamical equations of the diffusion.

In this work we solve a microscopical model of the heterogeneous medium which is compartmentalised by the thick and thin barriers that strongly impede diffusion and cause the transient-confinement of the diffusing particles. This choice of model is motivated by the ubiquity of measurements showing this so-called *hop diffusion* observed in biology^29–32,36–38^ and the *cage effect* in chemistry^42–44^. Non-Gaussian diffusion was observed together with these phenomena in multiple works, see the review^72^. In many other experiments the researchers were restricting their attention to only one of these two aspects of transport, which leaves an open and intriguing possibility of this link being much more prominent.

In our model the exponential tails of the probability density essentially stem from the lack of spatial correlations in the random medium. For media with more ordered microscopic structure, in particular with the periodically placed barriers, the observed probability density is expected to be much more Gaussian^41^ (see^68^ for theoretical discussion). However, if the barriers are placed independently, the distances between them are exponential. Any given temporarily trapped particle then has uniform distribution over its current domain, but the whole ensemble of particles scattered across the environment is a mixture of uniform distributions and exhibits exponential tails.

The simplicity of this argument makes it quite general, but it is not enough to specify the dynamics. For this we show how the local behaviour of the particle is equivalent to a classical heat transfer problem and then derive the escape times from the domains.

With the known domain sizes and transitions between them it becomes straightforward to interpret the “hops” of the *hop diffusion* as the jumps of a random walk with their distribution directly determined by the system’s geometry. Given this approximation, Monte Carlo simulations then show that it is indeed close to the original diffusion and preserves the non-Gaussian shape of the probability density bulk. Both are also the cases of non-Gaussian diffusion which at long times reaches Brownian limit with the same effective diffusivity.

This process is Fickian in both short and long timescales, but they differ by the diffusivity, which transitions from the initial “unhindered” short-time *D* to the lower $D_\text {eff}={\langle {\kappa _B}\rangle }D$; Such decay of diffusivity was reported in numerous experimental works^38^. The mean Biot number ${\langle {\kappa _B}\rangle }$ is determined by distances between barriers and their permeability, ${\langle {\kappa _B}\rangle } = \kappa {\langle {L}\rangle }$ and our results extends the analytical proofs for the systems with periodically placed barriers^68,69^. This link between micro- and macroscopic properties of the system may be verified experimentally by comparing the estimated diffusivity to the measured parameters of the barriers and their distribution in space.

It also causes the mean square displacement to be a convex function and the diffusion becomes subdiffusive in the transient time range of the diffusivity decrease. This is a well known phenomenon^72^, but our model also suggest that this behaviour is linked to the non-Gaussian probability density with exponential tails which may be observed in the same time regime. Subdiffusive non-Gaussian observations were reported in^13,42–44^. The derived leading behaviour of the probability density (36), formulas for kurtosis (25) and logarithm of the characteristic function (22) may be of use in analysing this type of experimental data.

The model which we consider is one-dimensional so one must be careful with applying it to systems with higher dimensionality. Nevertheless, it provides strong insights for the crucial aspects of the dynamics applicable to the less idealised cases: the random walk behaviour should be observed for more general systems divided into the localised, strongly confining domains. What changes are the domain sizes and escape times. But, during our analytical calculations of the non-Gaussianity measures and the probability density we introduced few subsequent simplifications to the model, removing the dependence of the environment, correlation and even approximating transition times. Yet, all the obtained random walks were remarkably similar, showing a surprising level of universality in our results.

It should be stressed that by this argument we actually establish not one, but three connections, each remarkable on its own: between systems with barriers and random walks, between random walks and *Brownian, yet non Gaussian diffusion*, and finally, between non Gaussian diffusion and systems with barriers. This approach opens new research possibilities in each of these areas, but also offers immediate scientific benefits: we manage to express the probability density of the displacements, non-Gaussianity measures and the effective diffusion coefficients using three parameters which describe the medium at microscopic level: in-domain diffusion coefficient, the average distance between barriers and their permeability. These relations form a valuable link between the molecular structure of the systems in question and the measurements of the non-Gaussian diffusion.

## Notation

- i.i.d. independent and identically distributed.
- $\overset{d}{=}$ equal in distribution, has a distribution.
- $${\mathcal {U}}nif(a,b)$$ uniform distribution, $$p_{{\mathcal {U}}nif(a,b)}(x) = {\varvec{1}}_{(a,b)}(x)/(b-a)$$.
- ${\mathcal {G}} am(\alpha ,\lambda )$ gamma distribution, $p_{{\mathcal {G}} am(\alpha ,\lambda )}(x) = \frac{\lambda ^\alpha }{\Gamma (\alpha )} x^{\alpha -1}\mathrm {e}^{-\lambda x}$; it has mean $\alpha /\lambda$.
- ${\mathcal {E}}xp(\lambda )$ exponential distribution, ${\mathcal {E}}xp(\lambda ) = {\mathcal {G}}(1,\lambda )$; it has mean $1/\lambda$ thus $\lambda$ is decay rate.
- ${\mathcal {L}}ap(\mu ,s)$ Laplace distribution, $p_{{\mathcal {L}}ap(\mu ,s)}(x) = \frac{1}{2s}\mathrm {e}^{-|x-\mu |/s}$; it has variance $2s^2$.
- $I_n(x),K_n(x)$ modified Bessel functions of the second kind.
